# Epidemiology of hip fracture: Worldwide geographic variation

**DOI:** 10.4103/0019-5413.73656

**Published:** 2011

**Authors:** Dinesh K Dhanwal, Elaine M Dennison, Nick C Harvey, Cyrus Cooper

**Affiliations:** Maulana Azad Medical College, New Delhi, India; 1 MRC Epidemiology Resource Center, University of Southampton, Southampton General Hospital, Southampton SO16 6YD, UK; 2 Botnar Research Center, Institute of Musculoskeletal Sciences, University of Oxford OX3 7LD, UK

**Keywords:** Epidemiology, geographic variation, hip fracture, incidence rate, osteoporosis

## Abstract

Osteoporosis is a major health problem, especially in elderly populations, and is associated with fragility fractures at the hip, spine, and wrist. Hip fracture contributes to both morbidity and mortality in the elderly. The demographics of world populations are set to change, with more elderly living in developing countries, and it has been estimated that by 2050 half of hip fractures will occur in Asia. This review conducted using the PubMed database describes the incidence of hip fracture in different regions of the world and discusses the possible causes of this wide geographic variation. The analysis of data from different studies show a wide geographic variation across the world, with higher hip fracture incidence reported from industrialized countries as compared to developing countries. The highest hip fracture rates are seen in North Europe and the US and lowest in Latin America and Africa. Asian countries such as Kuwait, Iran, China, and Hong Kong show intermediate hip fracture rates. There is also a north–south gradient seen in European studies, and more fractures are seen in the north of the US than in the south. The factors responsible of this variation are population demographics (with more elderly living in countries with higher incidence rates) and the influence of ethnicity, latitude, and environmental factors. The understanding of this changing geographic variation will help policy makers to develop strategies to reduce the burden of hip fractures in developing countries such as India, which will face the brunt of this problem over the coming decades.

## INTRODUCTION

Osteoporosis represents a major public health problem because of its association with low-energy trauma or fragility fractures. Hip fracture has been recognized as the most serious consequence of osteoporosis because of its complications, which include chronic pain, disability, diminished quality of life, and premature death. Osteoporotic hip fracture is an established health problem in the West and is increasingly recognized as a growing problem in Asia as per the Asian Audit Report, 2009.^1^ With rising life expectancy throughout the globe, the number of elderly individuals is increasing in every geographical region, and it is estimated that the incidence of hip fracture will rise from 1.66 million in 1990 to 6.26 million by 2050.^2^

Studies over the last few decades have demonstrated geographic variation in the incidence of hip fracture across continents as well as among different parts of a region. Incidence of hip fracture is highest in Sweden and North America, with almost seven-fold lower rates in Southern European countries.^3^ Hip fracture rates are also lower in Asian and Latin American populations. But as three-quarters of the world’s population live in Asia, it is projected that Asian countries will contribute more to the pool of hip fractures in coming years. It is estimated that by 2050 more than 50% of all osteoporotic fractures will occur in Asia.^2^ This variation in the distribution of hip fracture over different regions of the world demonstrate that genetic and environmental factors play a role in the etiology of hip fracture. It is therefore worthwhile to examine the geographic variations in hip fracture and speculate on the factors responsible for these differences. This review will serve as an update of the epidemiology of hip fracture worldwide, with special emphasis on the geographic variations and etiological factors. This review was conducted using the PubMed database. The keywords that were employed included hip fracture, incidence rate, geographic variation, osteoporosis, and epidemiology. The articles were chosen on the basis of 1) focus (studies that specifically focused on geographic variation in hip fracture); 2) language (studies that were in English); and 3) methods (studies that used statistical tests to examine hip fracture incidence rates).

## ASIA

Hip fracture rates are available from many countries across Asia, including from Singapore, Taiwan, Japan, Malaysia, China, and the Middle East. Unfortunately, only projected figures are available from India, which is second most populous country in the world. Studies on hip fracture incidence rates are available from Japan, particularly from the Tottori prefecture, a region representative of the Japanese population in terms of demographic and economic status.^4^ A recent survey (Hagino *et al*.) identified 851, 901, and 1059 patients with hip fracture (aged 35 years and older) in 2004, 2005, and 2006, respectively. The residual lifetime risk of hip fracture at 50 years of age was estimated to be 5.6% for men and 20% for women. The study concluded that in the Japanese population aged 35 years or older, the crude incidence of hip fracture was 244.8 per 100 000 person-years from 2004 to 2006 and the gender-specific incidence was 99.6 per 100 000 person-years for men and 368 per 100 000 person-years for women. When these incidence rates were compared with that from 30 years ago, the authors concluded that the incidence of hip fracture in the Japanese population is increasing. This increasing incidence is due to the increase in the population of the elderly in Japan over the last three decades.

The highest incidence of hip fractures from Asia has been reported from Singapore. A study by Koh *et al*. revealed that hip fracture rates from 1991 to 1998 (per 100 000) were 152 in men and 402 in women; this was respectively 1.5 and 5 times higher than corresponding rates in 1960s.^5^ Examined by ethnicity, since 1960, the main increase in hip fracture rates has been seen in Chinese and Malays, while the rates in the Indian ethnic group appear to have decreased. The factors responsible for these racial differences include differences in the demographic profile, body weight, physical activity, prevalence of cigarette smoking and alcohol consumption, calcium intake, and frequency of falls in the community in elderly.

In Korea, Lim *et al*. analyzed the incidence and cost of hip fracture from 2001 to 2004 using data from the Health Insurance Review Agency, Korea.^6^ In individuals over 50 years of age, the number of hip fractures in women increased from 250.9/100 000 persons in 2001 to 262.8/100 000 in 2004, a 4.7% increase. However, hip fractures in men decreased from 162.8/100 000 in 2001 to 137.5/100 000 in 2004, a 15.5% decrease. The direct medical care costs of hip fracture increased from $62 707 697 in 2001 to $65 200 035 in 2004, and the proportional cost of hip fractures in the national medical costs increased by 4.5% over 4 years (from 0.200% in 2001 to 0.209% in 2004). On analysis of the population-based data obtained from the whole country from 2001 to 2004, the incidence rate of hip fractures in women (but not in men) and its cost have increased in Korea. This gender difference in the distribution of hip fractures underlines the need for aggressive intervention in osteoporosis in elderly women.

In 1995, the incidence rates of hip fracture from Hong Kong were 110/100 000 in women and 50/100 000 in men as per data from public hospitals.^7^ Secular trends on hip fracture from Hong Kong suggest that over the last three decades the age-specific incidence increased 2.5-fold in women and 1.7-fold in men. The incidence rates were found to similar to those seen in the Wessex health region of UK.^8^ In Beijing, China, hip fracture incidence were calculated from admissions to 76 city hospitals between 1988 and 1992.^9^ It was presumed that all the fracture cases from Beijing go to these public hospitals only. Based upon the 1990 China census, age-standardized rates of hip fracture were 87/100 000 for women and 97/100 000 for men. These data further demonstrate that from 1988 to 1992, the rates in Beijing increased by 34% in women and 33% in men.

Maximum data from the Middle East is available from Iran from the Iranian Multicenter Study on Accidental Injuries.^10^ This study reported age-standardized incidence rates of hip fracture of 127.3/100 000 person-years in men and 164.6/100 000 person-years in women, which is much lower than the rates reported from any of the Western countries, including the US. Smaller studies are available from Kuwait and show similar results.^11^

## LATIN AMERICA

Limited data is available from South American countries. Reira-Espinoza recently reviewed data from Latin America.^12^ In a study published from Mexico in 2005, the annual rate of hip fracture in the two public health care systems were 169 in women and 98 in men per 100000 person-years.^13^ These fracture rates are similar to that reported from the southern countries of Europe. Recently, hip fracture incidence has been reported from Rosario, Argentina.^14^ The annual incidence among inhabitants over the age of 50 years was 290/100 000 (405/100 000 for women and 137/100 000 for men; female/male ratio: 2.96). In the population over 65 years of age, the incidence was 646/100 000 and 345/100 000, respectively. These values are similar to the values reported in people older than 65 years in the US. Reports from Venezuela have shown that the incidence of hip fracture in women over 50 years was 98/100 000 and in men 37/100 000. With an increasing population of elderly individuals in this part of the world, as well as limited health care resources, clinicians and health authorities will face an enormous challenge in the coming years in Latin America.

## AFRICA

A few studies are available from Africa on the incidence of hip fracture. Osteoporosis and fragility fractures are believed to be uncommon in Africa. Zebaze *et al*. conducted a study in Cameroon by documenting all patients aged 35 years and older admitted to the two main urban hospitals in Cameroon over a 2-year period with a diagnosis of fracture.^15^ Using the 1997 estimates of the population, the incidence of low-energy trauma fractures at the hip (per 100 000 persons over 35 years) were 57.1 in women and 43.7 in men. Similar low fracture rates have been reported from Morocco in 2005.^16^ Based on these two fairly well-conducted studies from Africa it is difficult to make a general statement about hip fracture incidence from Africa, but it seems that as in American blacks, the hip fracture rates in the African population are much lower than in the western population.^17^

## NORTH AMERICA

Hip fracture rates among the US population are the highest in the world. Melton *et al*. and Ho *et al*. have reported age-standardized annual incidence of hip fracture per 100 000 as 201 and 197 in men and 511 and 553 in women, respectively.^17^ A recently published study has reported the secular trends in Minnesota in the US over the period from 1928 to 1992.^18^ This study looked at all proximal femur fractures that occurred among residents during the 65-year period. The annual age-adjusted incidence rates among women rose rapidly until 1950, only to fall slowly thereafter. Age-adjusted rates in men rose more steadily before beginning a downturn after 1980. Incidence rates rose exponentially with age in both men and women. Another study from the US used the national hospital discharge survey (which covers 0.6% of all patient discharges) to analyze secular trends in the white population from 1970 to 1983.^19^ An overall increase of 9.3% in age- and sex-adjusted hip fracture incidence rates was observed over the 14-year period. A second study using the same database looked at age-specific rates between 1965 and 1993.^20^ Hip fractures increased linearly for males in the age-groups of 80–84 years and ≥85 years, but for women and young men the rates did not change significantly over this time period. There was an overall improvement in the hospital survival rates in men aged over 85 years and females over 75 years of age.The Framingham study, a population-based cohort study conducted from 1948 to 1996, found that compared with women born by 1900, hip fracture incidence was 1.2 times and 1.4 times greater among women born from 1901 to 1910 and from 1911 to 1921, respectively.^21^ This study demonstrates an important birth cohort effect over this period, consistent with studies conducted in England^22^ and Finland.^23^ A Californian study looked at hip fracture rates between 1983 and 2000, with special attention paid to the Hispanic population, the largest and fastest-growing ethnic minority in the US.^24^ Hip fractures were identified using the annual hospital patient discharge database. Among non-Hispanic white women in California, the standardized annular hip fracture rates for those ≥55 years fell steadily over the past two decades by 0.6% per year in women (and 0.5% in men). No significant change occurred among black or Asian women migrants to the US. By contrast, the annual fracture rates amongst Hispanic women increased by 4.9% per year (and by 4.2% per year in the men). This supports the hypothesis that residence in early life has a much greater association with variation in hip fracture rates that does current region of residence. Another explanation may be that Hispanic men and women have been shown to partake in less physical activity and are more likely to have nutritional deficits than non-Hispanic whites. In a recent study by Brauer and co-workers (2009), it was concluded that in the US, hip fracture rates and subsequent mortality among persons ≥65 years of age are declining and that the comorbidities among patients with hip fractures have increased.^25^ This group looked at the data from a 20% sample of Medicare claims from 1985–2005 in patients ≥65 years. The annual mean number of hip fractures was 957.3/100 000 for women and 414.4/100 000 for men. The age-adjusted incidence of hip fracture increased from 1986 to 1995 and then steadily declined from 1995 to 2005. Leslie *et al*. have recently published data from Canada in a population-based study and made international comparisons.^26^ According to this group, during 2000–2005, 147 982 hip fractures were identified. The age-adjusted fracture incidence was 86.4/100 000 in women and 53./100 000 in men. On comparison with data from the US, the overall fracture rate in Canadian women was 30% lower than in US women in 2001 and 26% lower than in German women in 2004. Canadian men showed similar overall hip fracture rates as American men prior to age 80 years but a 26% lower rate after 80 years of age.

## EUROPE

The majority of studies detailing hip fracture rates have been performed in the last five decades. Scandinavia has the highest reported incidence of hip fracture worldwide. There are a large number of studies looking at incidence rates as well as secular trends in this geographically northern region. The incidence rates vary from North to South Europe, with the highest being in Sweden and Norway and the lowest in France and Switzerland. From Norway, the reported age-standardized annual incidence rate of hip fracture is 920/100 000 in women and 399.3/100 000 in men and that in Switzerland is 346/10000 and 137.8/100 000 in women and men, respectively. A study on secular trends from Uppsala, Sweden, (1965–1980) showed an annual increase of 2.2% for age- and sex-adjusted hip fracture rates, which increased from 430/100 000 in 1965 to 650/100 000 in 1980.^27^ The age-specific incidence increased especially in the group aged ≥85 years, in whom fractures of the femoral neck were three times more common and trochanteric fractures four times more common in 1980 than in 1965. Studies from Malmo, Sweden, showed an exponential increase in hip fracture incidence from 1950 to 1985 in both men and women over age 50, increasing from an annual age-adjusted incidence of 150–390/100 000 in men and 300–830/100 000 in women.^28^ Increases were seen for both trochanteric and cervical fractures. However, the most recent data from Malmo from 1992–1995 shows that this fracture rate is now steady, in line with many of the Northern American studies.^29^ If the data is broken down according to fracture subtypes, however, the incidence of cervical fractures had decreased (210–170/100000 in men and 420–410/100000 in women), whereas there was still a small rise in trochanteric fractures (180–190/100000 in men and 410–440/100000 in women).

The incidence of hip fractures in Oslo, Norway, between 1978 and 1997 was assessed using electronic diagnosis registers.^30^ The age-adjusted fracture rates per 10000 were 118.0 and 44.0 in 1996/97, 124.3 and 44.9 in 1988/89, and 104.5 and 35.8 in 1978/79 for women and men, respectively, indicating that the incidence of hip fractures in Oslo has not changed significantly during the last decade. Denmark used its national patient register to look at hip fracture incidence between 1987 and 1997 in Viborg County.^31^ The incidence of age-adjusted first hip fractures increased significantly by 18 and 8 per 100 000 per year for women and men, respectively; peri-trochanteric fractures increased by 10 per 100 000 per year There are a number of studies from Finland, all using the Finnish National Hospital discharge register. The first study drew its hip fracture data from the entire >50-year-old population. Between 1970–997, the age-adjusted hip fracture rate increased from 292/100 000 to 467/100 000 in women and from 112/100 000 to 233/100 000 in men.^23^ In a further follow-up study using the same population from 1997–2004, the age-adjusted incidence fell nationally by 2.4% annually in women and 0.9% in men in 2004.^32^ A smaller study in central Finland between 1992–2003 showed that the age-adjusted rates increased considerably from 2.0 per 1000 person-years to 3.9 per 1000 person-years in men and from 2.8 per 1000 person-years to 5.6 per 1000 person-years in women.^33^ There is no obvious explanation why central Finland should have increasing rates compared to the rest of the country. One possibility is that only two time points were examined in this study, whereas most other studies have examined the secular trends; it is possible that a sharp rise in the rates may have occurred during the early part of the study period and then stabilized.

Overall, the epidemiological data from Scandinavia indicates that although early studies (dating to the1950s) described an increase in hip fracture incidence, the rates appear to have fallen in the most recent periods. The decline in fracture rate appears to have occurred several years later in Finland compared to Sweden and Norway. The rise in the incidence of hip fracture in Finland from the early 1970s until the late 1990s has been followed by declining fracture rates. The exact reasons for this are unknown, but a cohort effect toward a healthier aging population and increased average body weight and improved functional ability among elderly Finns cannot be ruled out as possible causes.^34^

Data from central Europe includes studies from the UK, Netherlands, Germany, Switzerland, Austria, and Hungary. The highest number of studies comes from the UK. The first study on this subject assessed hip fracture data from the hospital inpatient enquiry for England and Wales between 1968–78.^35^ In this study, the age-specific rates increased steadily in women by 61% and in men by 73% until 1979. No further increases occurred in either sex up to 1985. The Oxford Record Linkage Study which looked at the period 1968–1986 found a similar pattern, although there was a more continuous trend.^22^ Age, cohort, and period modelling were used in this study to look at the incidence rates. There was a clear cohort effect in both the studies and the difference in incidence rates was apparent from births in 1883 to 1917 in addition to the age. A birth cohort effect was confirmed in subsequent analyses of the Framingham data, showing that in progressive birth cohorts from 1887–1921 there were age-specific increases in fracture incidence rate ratios up from 1.0 to 1.2–1.4 in women and 1.0 to 2.0 in men.^21^ The most recent study from the UK looked at hospital episode statistics from 1989–1998.^36^ Age-standardized incidence rates increased by 32% in women and 38% in men up to 1991–92 and thereafter remained stable. In the Netherlands, data from the Dutch Medical Registry shows that between 1972–1987 the age-adjusted incidence of hip fractures rose linearly from 479/100 000 to 669/100 000 per year in women and from 198/100 000 to 308/100 000 per year in men aged ≥65 years.^37^ In a later study conducted between 1986 and 2002 using the Dutch Medical Registry, the age incidence of hip fracture increased linearly from 1986–1993 in patients over 45 years;^38^ after this the incidence decreased by 0.5% annually until 2002. In Germany, between 1995 and 2004 (in a study using the national hospital discharge register) the age- and sex-adjusted hip fracture incidence increased by 0.5% per year in women and 0.7% per year in men.^39^ In women aged ≥40 years there was a tendency for a decrease up to the age of 74 years, but there was a pronounced increase in patients over 75 years. Interestingly, the increase was significantly higher in Eastern Germany compared to Western Germany, particularly in the older age-groups, which suggests that the differences between the East and West decreased over time.

Hip fracture trends in Geneva, Switzerland, between 1991 and 2000 have been studied using computerized medical records from the main hospital. The study found a significant decline of 1.4% annually for age-adjusted rates in women, but the rates remained stable in men.^40^ In neighbouring Austria, between 1994 and 2006, after adjustment for age and sex, the incidence rates rose from 471/100 000 to 567/100 000 per year in men and from 637/100 000 to 759/100 000 per year in women.^41^ This rise is predominantly accounted for by the rise seen in patients aged over 80 years. Hip fracture rates in Hungary are available for the period 1999–2003 from the National Health Insurance Fund database. This database covers the whole population of Hungary.^42^ The age-adjusted incidence of hip fracture was 430/100 000 in women and 223/100 000 in men; these rates have remained stable over this period. A lesser number of studies are available from southern Europe; two of these have been chosen to represent hip fractures from this region. An Italian study looked at the incidence of hip fracture in the county of Sienna from 1980–1991 using records from the orthopedic departments of various hospitals.^43^ During this 12-year period, the temporal trend rose linearly in men from 57.5/100 000 person-years to 108.9/100 000 person-years: a 7.4% annual increase. In females, no significant trend was observed. The overall incidence rate during this period was 157/100 000, much lower than that in northern or central European countries. Another study from Spain looked at hip fracture trends in northern Spain between 1988 and 2002 using clinical records from all hospitals in the region of Cantabria.^44^ Whilst the crude hip fracture incidence increased during this period, no significant changes were noticed following adjustment for age. Neither was there a noticeable trend in age-specific incidence rates. The crude rate increased mainly among with a more noticeable rise in cervical fractures as opposed to trochanteric fractures.

## OCEANIA

Studies of hip fracture epidemiology have also been performed in both New Zealand and Australia and the fracture incidence rates are comparable to that seen in the Caucasian population in Europe and North America. A study from New Zealand between 1950–1987 looked at nationwide hip fractures in those aged ≥65 years.^45^ A disproportionate increase in the number of fractures in relation to the increase in population size was observed. The group at highest risk were women over the age of 85 years. A later study from 1988–1999, using data from the New Zealand Health Information Service, found that the numbers of males and females aged ≥65 years with hip fracture did not meet the predictions.^46^ In fact, age-specific hip fracture rates dropped significantly for females in all the age bands tested and have remained unchanged in men.

In Australia there have been two major studies. The first, by Chang *et al*., looked at fracture rates between 1989 and 2000 in Dubbo (a semi-urban city 400 km Northwest of Sydney).^47^ They showed that there was a significant reduction in the overall fracture incidence rate by 4% per year in women and 6% per year in men. There was no significant change in the number of hip fractures over the same period; however, the total number of hip fractures seen in this study was relatively low and hence it was underpowered. A second study looked at hospital admissions for hip fracture in New South Wales between 1990–2000. Whilst the crude incidence rose, age-adjusted rates remained unchanged at around 130/100 000 person-years in men and 390/100 000 person-years in women.^48^ Women aged 65–75 years were the only age-specific group with a 1% decline in annual incidence of hip fracture, the incidence in the other age-groups remaining unchanged during the period. What might explain the variation in hip fracture incidence in different regions of world?

**Figure 1:**
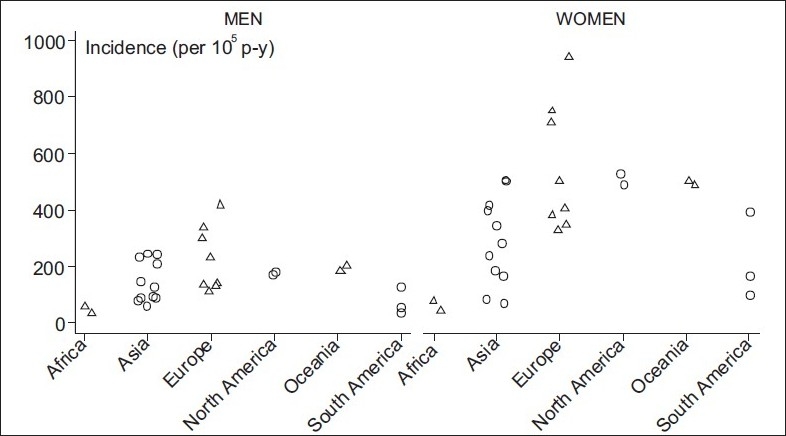
Hip fracture per 100 000 person-years in different continents

Age is the main risk factor for hip fractures. The incidence of hip fracture increases exponentially with age in both genders. In females younger than 35 years, the incidence is 2/100 000 person-years, whereas it is 3032/100 000 person-years in women older than 85 years. In men, the corresponding rates are 4 and 190 per 100 000 person-years. Most hip fractures occur in the elderly: 52% after the age of 80 years and 90% after the age of 50 years.^2^ The decline in bone mineral density and the increase in frequency of falls in elderly people are mainly responsible for this high incidence of hip fractures. Only 1% of falls lead to a hip fracture, but 90% of these fractures is related to a fall from standing height or less. To investigate this issue, Schwarts *et al*.^49^ carried out a cross-national study of hip fracture in five geographic areas – Beijing, China; Budapest, Hungary; Hong Kong; Porto Alegre, Brazil; and Reykjavik, Iceland – during the years 1990–1992. Cases of hip fracture among women and men of ≥20 years were identified using hospital discharge data in conjunction with medical records, operating room logs, and radiology logs. Estimated rates varied widely, with Beijing reporting the lowest rates (45.4/100 000 in men and 39.6/100 000 in women) and Reykjavik the highest rates (men: 141.3/100 000; women: 274.1/100 000). The rates were higher for women than for men in all areas except Beijing. The study demonstrated large differences in hip fracture incidence rates, with age-adjusted incidence rates in women being 6 times higher and in men over 3 times higher in Reykjavik compared with Beijing. The results of this study indicate the substantial limitations of relying on the hospital discharge data alone to estimate hip fracture incidence rates; however, the error found in the discharge lists is smaller than the large international variation found. The study concluded that the differences reported among countries reflect genuine variation in the hip fracture incidence rates. The influence of ethnicity on risk of osteoporotic fractures was analyzed by Ellaine *et al*. from our center (unpublished data). The rates vary considerably according to the geographic area and race and may vary widely within the same country and within populations of a given sex and race [Figure 1]. In Europe, hip fracture rates vary by as much as 7-fold between countries. In general, people who live in latitudes far from the equator seem to have a higher incidence of fracture. The highest rates of hip fracture are seen in Caucasians living in northern Europe, especially Scandinavians. A study from 1989 found that the age-adjusted 1-year cumulative incidence of hip fracture in Norway was 903/100 000 for women and 384/100 000 for men.^30^ The rates are intermediate in Asia, China, and Kuwait and lowest in black populations. While studies in central Norway suggest a stabilization in fracture rates in recent years, a Californian study published in 2004 reported a doubling of hip fracture rates in Hispanics, while no significant change occurred among black or Asian men or women. In many cases, the lower incidence rates seen in the developing countries can be partially explained by the lower life expectancy; in Latin America only 5.7% of the population is over 65. Reduced longevity may also be the explanation for the low fracture rates observed in Morocco. Genetic factors may play an important role in the etiology of hip fracture, as also environmental factors. However, those factors that have been studied so far – such as alcohol consumption, smoking, activity levels, obesity, and migration status – have not explained these trends. Further research is clearly needed to explain these important environmental factors. Diseases associated with secondary osteoporosis and with increased risk of falling are an important cause of hip fractures, but more so in men than in women.

The incidence of hip fracture varies among different countries and populations [Table 1 and Figure 1]. Rates are higher in Scandinavia than in Western Europe and Oceania. A north-south gradient in age-standardized risk is found in Europe and US, with higher rates in north. The age-adjusted increase in incidence that has been observed in several countries over the last 50 years appears to have levelled off in some of these countries, especially in Europe and the US. The incidence increases with poor economic status, reduced winter sunlight, and water fluoridation. Fractures occur more commonly in the winter season due to altered neuromuscular coordination and vitamin D deficiency. The incidence of hip fracture is significantly lower in black and Asian people. These geographical and racial differences could be explained as being due to the reduced life expectancy in Asian countries, the genetic background, and high physical activity. No studies are available from the Indian subcontinent regarding hip fracture incidence. With changing the demographic profile of Indian population, we are going to witness a sharp rise in hip fractures over the next three decades.^2^ Therefore, there is urgent need to carry out epidemiological studies from India and other parts of Asia to allow health administrators to plan policies for prevention of hip fracture in elderly population.

**Table 1:** Age-standardized hip fracture rates (per 100 000 population) across different continents

Continent	Country	Men	Women
	Morocco, Rabat	57.7	79.9
	Cameroon	43.7	52.1
Asia	China, Beijing	87	97
	China, Shenyang	101.3	80.9
	Korea	137	262
	Iran	127.3	164.6
	Malaysia	87.4	212.5
	Japan, Tottori	107.3	297.3
	Japan	99.6	368
	Kuwait	216.6	316
	Singapore	152	402
	Hong Kong	193	484.3
	Hong Kong	50	110
	Taiwan	233.4	496.8
South America	Mexoci	98	169
	Brazil, Sobral	59.3	168.4
	Argentina	137	405
	Venezuela	37	98
Europe	Switzerland	137.8	346
	Former East Germany	137.8	354.7
	Former West Germany	154.5	399.4
	England	143.6	418.2
	Greece	201.7	469.9
	Sweden	302.7	709.5
	Norway,	352	763.6
	Norway, Oslo	399.3	920.7
	Austria	567	759
	Hungary	223	430
	The Netherlands	308	669
North America	United States, Minnesota	201.6	511.5
	United States	197.2	553.5
Oceania	New Zealand, Maori	197	516
	Non-Maori	288	827
	New South Wales	191.8	475.1
	Australia	187.8	504.2
	Australia	130	390
